# An Adaptive Zero Velocity Detection Algorithm Based on Multi-Sensor Fusion for a Pedestrian Navigation System

**DOI:** 10.3390/s18103261

**Published:** 2018-09-28

**Authors:** Ming Ma, Qian Song, Yang Gu, Yanghuan Li, Zhimin Zhou

**Affiliations:** School of Electronic Science, National University of Defense Technology, Changsha 410073, China; songqian@nudt.edu.cn (Q.S.); guyang@nudt.edu.cn (Y.G.); liyh_gfkd@163.com (Y.L.); kdzhouzhim@163.com (Z.Z.)

**Keywords:** pedestrian navigation system, ZUPT, zero velocity detection, adaptive threshold, stairs recognition

## Abstract

The zero velocity update (ZUPT) algorithm is an effective way to suppress the error growth for a foot-mounted pedestrian navigation system. To make ZUPT work properly, it is necessary to detect zero velocity intervals correctly. Existing zero velocity detection methods cannot provide good performance at high gait speeds or stair climbing. An adaptive zero velocity detection approach based on multi-sensor fusion is proposed in this paper. The measurements of an accelerometer, gyroscope and pressure sensor were employed to construct a zero-velocity detector. Then, the adaptive threshold was proposed to improve the accuracy of the detector under various motion modes. In addition, to eliminate the height drift, a stairs recognition method was developed to distinguish staircase movement from level walking. Detection performance was examined with experimental data collected at varying motion modes in real scenarios. The experimental results indicate that the proposed method can correctly detect zero velocity intervals under various motion modes.

## 1. Introduction

With the rapid development of micro-electro-mechanical system (MEMS) technology, MEMS inertial sensor-based pedestrian navigation systems play an important role for personal positioning indoors [1,2,3,4,5,6]. However, low-cost inertial sensors suffer from sensor drift and due to the integrative nature of the inertial navigation systems (INS), any small bias will accumulate and grow with time boundlessly [7,8]. Fortunately, a foot-mounted INS aided by the zero velocity update (ZUPT) technique has shown its ability to suppress navigation errors [9,10,11,12,13]. The performance of the ZUPT technique highly relies on the accuracy of zero velocity detection. A range of detectors that detect the zero velocity intervals from the output of the accelerometers or gyroscopes have been proposed [14,15,16]. In Reference [17], the zero velocity detection was formalized as a binary hypothesis testing problem and four likelihood ratio test (LRT) detectors were proposed. The detectors provided good performance at slow speeds (approximately 3 kph) and normal speeds (approximately 5 kph). The methods mentioned above are all threshold-based. These threshold values could differ significantly when a person is walking and running. The outputs of inertial sensors tend to become large and the zero velocity intervals tend to become shorter when a person is running. If the predefined threshold values are too small, then the zero velocity intervals cannot be detected when a person is running. 

To solve this problem, extensive research has been performed. In Reference [18], an adaptive stance-phase detection method was proposed. In this method, an additional accelerometer was attached to the chest and the difference of maximum acceleration change extracted from the chest acceleration was adopted to update the corresponding threshold for zero velocity detection. In Reference [19], the magnitude peak of *y*-axis gyroscope was used to update the threshold based on the pre-defined threshold function. In Reference [20], an algorithm based on a Markov model was developed which only uses the segmentation of the *y*-axis gyroscope outputs instead of the three-axis output. The method used in References [18,19,20] showed good performance under walking and running modes. However, none of these methods explored zero velocity detection when a person ascends or descends stairs. In Reference [21], a detection method was constructed using the variations in speed during a gait cycle. This method performed well under stair descending and ascending modes. The main limitation of this method was that the zero velocity detection of previous steps can affect the detection of current step. Extra non-inertial sensors have been used to aid zero velocity detection, such as magnetometers [7], radio frequency sensors [22] and electromyography sensors [23]. Although using extra sensors might improve detection accuracy, these methods require expensive and specialized equipment.

In this paper, we propose a novel zero velocity detection method based on multi-sensor fusion. The measurements of accelerometer, gyroscope and pressure sensor were combined to detect the zero velocity intervals. Then, an adaptive threshold and stairs recognition were developed to improve the accuracy and reliability of detection. The new detector provided good performance under various motion modes. 

## 2. Zero Velocity Detection

### 2.1. Problem Description

**Figure 1:**
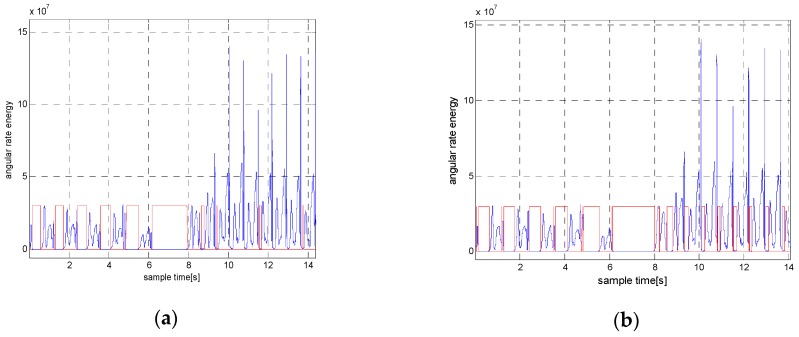
The zero velocity detection results of the SHOE method when a pedestrian is walking and running. (**a**) The detection result using a small threshold. (**b**) The detection result using a large threshold. The red line denotes the stationary state and moving state. Small values indicate the moving state, while large values indicate the stationary state.

In most methods, detection thresholds must be assigned prior to detection. These thresholds differ significantly under various motion modes, such as walking, running and ascending stairs. If the threshold values are too large, false zero velocity intervals could be detected. Conversely, if we choose the threshold to be too small, the zero velocity intervals cannot be detected when a pedestrian is running. We show this in Figure 1. In general, the performance of the stance hypothesis optimal detection (SHOE) algorithm in Reference [17] is relatively better than many other methods. Thus, we used this method to verify that it is difficult to find a widely applicable threshold. Figure 1a depicts that although the SHOE method performs well when the person is walking, only half of the zero velocity intervals are detected during running with a small threshold. Figure 1b shows that the SHOE method can detect most of the zero velocity intervals of running using large threshold. However, it produces false detection for walking and running targets, which degrades the accuracy of positioning.

**Figure 2:**
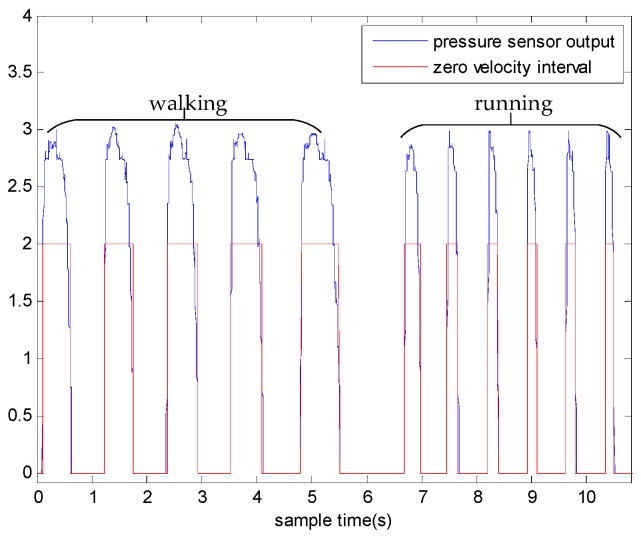
The measurements of the pressure sensor during walking and running.

**Figure 3:**
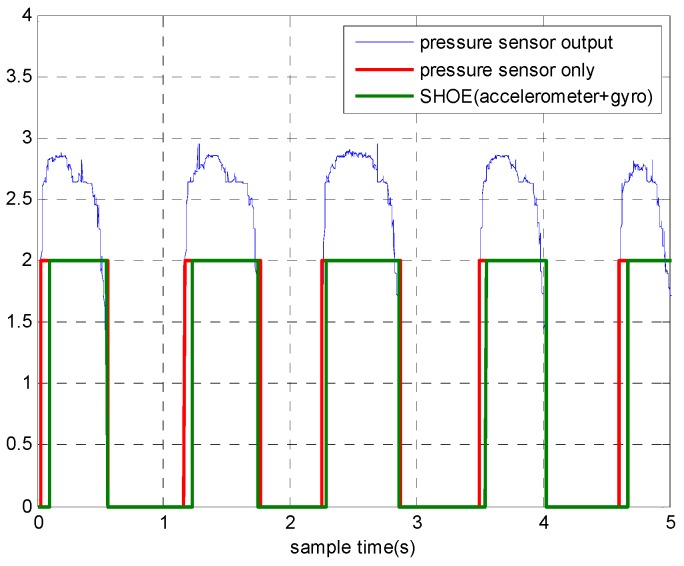
The detection results of zero velocity intervals.

To solve this problem, we propose a novel zero velocity detection approach based on the multi-sensor fusion. Besides accelerometers and gyroscopes, we also adopted pressure sensors to assist detection. Figure 2 shows the output of the pressure sensor when the person is walking and running. As the output of pressure sensor is almost independent of the motion mode, we considered only using a pressure sensor to detect zero velocity intervals at the beginning of the research. However, the detection accuracy of using pressure sensor was not satisfactory. The results of Reference [24] show that the SHOE detector outperformed the pressure sensor-based detector. We also conducted experiments to compare the performance of the two detectors. The pedestrian walked along a square trajectory, 6 m long, for eight loops. The experiment was repeated for 10 times and 10 datasets were collected. The detection results are shown in Figure 3 and it can be observed that the zero velocity intervals detected by the pressure sensor-based detector always started earlier than those detected by the SHOE detector. The mean positioning errors of the SHOE detector and the pressure sensor-based detector are 0.8 and 1.5 m, respectively. These results indicate that only using the pressure sensor for detection is not an optimal choice. Hence, we propose a detector based on multi-sensor fusion and the proposed detector is introduced as follows.

Assume that the INS assembled with an IMU (three-axis accelerometers and three-axis gyroscopes) and a pressure sensor, has been installed into the heel of a foot. Let $m_{k}\in {\mathbb{R}}^{7}$ denote the output from the IMU and pressure sensor.

(1) $$m_{k}={\left[\begin{array}{ccc} m_{k}^{a} & m_{k}^{\omega } & m_{k}^{p} \end{array}\right]}^{T}$$

where $m_{k}^{a},m_{k}^{\omega }\in {\mathbb{R}}^{3}$ denote the measured specific force vector and the angular rate vector at time k, respectively. $m_{k}^{\rho }\in \mathbb{R}$ represents the measurement of the pressure sensor. The purpose of the zero velocity detection is to decide whether the IMU is stationary or moving between the time instants n and n+N−1, given the measurement sequence $q_{n}={\left\{m_{k}\right\}}_{k=n}^{n+N-1}$. The false-alarm probability (i.e., the probability of deciding that the IMU is stationary when it is not) should be kept low. This is because the effect of imposing a false zero velocity constraint on the positioning system can greatly deteriorate the positioning accuracy. Furthermore, as imposing the true zero velocity constraints is the key to eliminating cubic error accumulation, the probability of detecting the zero velocity intervals should be maximized given a certain false-alarm probability. Mathematically, the zero velocity detection problem can be formalized as a binary hypothesis testing problem. The two hypotheses, $H_{0}$ and $H_{1}$, can be defined as follows.

(2) $$\begin{array}{l} H_{0}:\;\mathrm{IMU}\;\mathrm{is}\;\mathrm{non}-\mathrm{stationary}\\ H_{1}:\;\mathrm{IMU}\;\mathrm{is}\;\mathrm{stationary} \end{array}$$

The performance of the detector is determined by the probability of detection ($P_{D}=p(H_{0}|H_{1})$) and the false-alarm probability ($P_{\mathrm{FA}}=\;p(H_{1}|H_{0})$). Based on the Neyman–Pearson theorem, for a given $P_{\mathrm{FA}}=\alpha$, decide on $H_{1}$ to maximize $P_{D}$ if

(3) $$T\left(q_{n}\right)=\frac{p\left(q_{n};H_{1}\right)}{p\left(q_{n};H_{0}\right)}>\gamma$$

$p\left(q_{n};H_{1}\right)$ and $p\left(q_{n};H_{0}\right)$ denote the probability density functions of the observations for the hypothesis $H_{1}$ and $H_{0}$, respectively and the threshold γ is determined by

(4) $$P_{\mathrm{FA}}=\int_{\left\{q_{n}:T\left(q_{n}\right)>\gamma \right\}}p\left(q_{n};H_{0}\right)dq_{n}=\alpha \;$$

The test in Equation (3) is referred to as the likelihood ratio test (LRT). 

### 2.2. Sensor and Signal Model

The probability density functions of the observed data depend on the true signal and the sensor noise. Therefore, the signal and sensor model should be specified to find probability density functions. For IMU sensors, the IMU measurement can be described as follows.

(5) $$m_{k}=s_{k}+v_{k}$$

where

(6) $$\begin{array}{c} s_{k}={\left[\begin{array}{ccc} s_{k}^{a} & s_{k}^{\omega } & s_{k}^{\rho } \end{array}\right]}^{T}\\ v_{k}={\left[\begin{array}{ccc} v_{k}^{a} & v_{k}^{\omega } & v_{k}^{\rho } \end{array}\right]}^{T} \end{array}$$

here $s_{k}^{a}\in {\mathbb{R}}^{3}$, $s_{k}^{\omega }\in {\mathbb{R}}^{3}$ and $s_{k}^{\rho }\in \mathbb{R}$ denote the IMU-experienced acceleration, the angular rate and pressure at time k, respectively. Moreover,$v_{k}^{a}\in {\mathbb{R}}^{3}$, $v_{k}^{\omega }\in {\mathbb{R}}^{3}$ and $v_{k}^{\rho }\in \mathbb{R}$ represent the measurement noise of the accelerometer, gyroscope and pressure sensor, respectively. Here, we assume that the measurement noise is independent, identically-distributed white Gaussian noise with the covariance matrix

(7) $$C=E\{v_{k}v_{k}^{T}\}=\left[\begin{array}{ccc} \sigma_{a}^{2}I_{3} & 0_{3} & 0_{3\times 1}\\ 0_{3} & \sigma_{\omega }^{2}I_{3} & 0_{3\times 1}\\ 0_{1\times 3} & 0_{1\times 3} & \sigma_{\rho }^{2} \end{array}\right]$$

where $I_{3}$ denotes an identity matrix of three orders, $0_{3}$ denotes a zero matrix of three orders and $0_{3\times 1}$($0_{1\times 3}$) denotes a zero matrix of size 3×1(1×3). $\sigma_{a}^{2}\in \mathbb{R}$ and $\sigma_{\rho }^{2}\in \mathbb{R}$ denote the noise variance of the accelerometer, gyroscope and pressure sensor, respectively. Ε{⋅} and ${\left\{\cdot \right\}}^{T}$ denote the expectation operation and transpose operation, respectively. 

Under the hypothesis $H_{0}$, modeling the signal in a consistent way is difficult. Under the hypothesis $H_{1}$, gravitation acceleration is the only specific force observed by the accelerometer and the angular rate experienced by the gyroscope is zero. Moreover, the measurements of the pressure senor will reach the maximum value ($\rho_{\max}$) when the IMU is stationary. In other words, for the two hypotheses, the signal fulfills the restraint conditions

(8) $$\begin{array}{l} H_{0}:\exists k\in \Omega_{n},\;s.t.\;s_{k}^{a}\neq gu_{n}\;\mathrm{or}\;s_{k}^{\omega }\neq 0\;\mathrm{or}\;s_{k}^{\rho \prime }\neq \rho_{\max}\\ H_{1}:\forall k\in \Omega_{n},\;\mathrm{then}\;s_{k}^{a}=gu_{n}\;\mathrm{and}\;s_{k}^{\omega }=0\;\mathrm{and}\;s_{k}^{\rho \prime }=\rho_{\max} \end{array}$$

**Figure 4:**
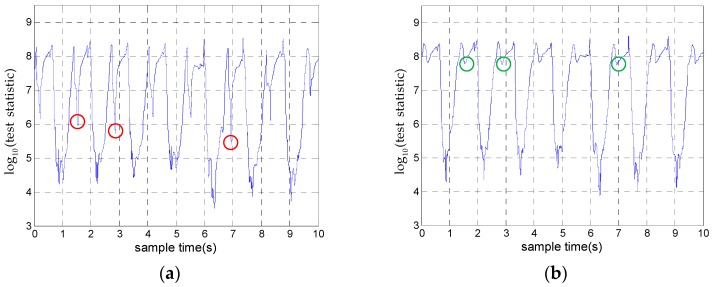
The test statistics of zero velocity intervals: (**a**) The test statistics of the SHOE detector. (**b**) The test statistics of the proposed detector.

Here, $u_{n}\in {\mathbb{R}}^{3},\left\|u_{n}\right\|=1$ and $\Omega_{n}=\left\{\ell \in \mathbb{N}:\;n\leq \ell <N-1\right\}$. Using the mathematical method in Reference [17], we can derive the proposed detector as follows:

(9) $$\frac{1}{N}\sum_{k\in \Omega_{n}}\left(\frac{1}{\sigma_{a}^{2}}{\left\|m_{{}_{k}}^{a}-g\frac{{\bar{m}}_{n}^{a}}{\left\|{\bar{m}}_{n}^{a}\right\|}\right\|}^{2}+\frac{1}{\sigma_{\omega }^{2}}{\left\|m_{{}_{k}}^{\omega }\right\|}^{2}+\frac{1}{\sigma_{\rho }^{2}}{\left\|m_{{}_{k}}^{\rho }-\rho_{\max}\right\|}^{2}\right)<\gamma^{\prime }$$

where γ′=−2(lnγ)/N. If the Equation (9) is satisfied, we choose the hypothesis that the IMU is stationary. Compared with the SHOE detector, the test statistic of the proposed detector adds the parameter ${\left\|m_{{}_{k}}^{\rho }-\rho_{\max}\right\|}^{2}/\sigma_{\rho }^{2}$. During the zero velocity intervals, this parameter is almost zero and it is a big value during motion intervals. Thus, this parameter makes the difference of statistics between motion intervals and zero velocity intervals more obvious. Figure 4a,b shows the test statistics of the SHOE detector and the proposed detector, respectively. In Figure 4a, the statistics marked by red circles are easily detected incorrectly as zero velocity intervals. Figure 4b shows the statistics after adding the parameter ${\left\|m_{{}_{k}}^{\rho }-\rho_{\max}\right\|}^{2}/\sigma_{\rho }^{2}$ and the statistics marked by green circles correspond to the statistics marked by the red circles in Figure 4a, which are obviously larger. This shows that the proposed detector can reduce the false alarm detection probability.

## 3. Adaptive Threshold

As shown in Equation (9), the proposed detector determines the zero velocity interval based on the comparisons between the threshold ($\gamma^{\prime }$) and the test statistic. By integrating the measurements of the accelerometer, gyroscope and pressure sensor, the proposed detector can distinguish between the swing and the stance phase more accurately. Although the proposed detector can detect zero velocity intervals for both walking and running modes by using an appropriate threshold, the detection precision of each gait cycle will be different. Moreover, for a single motion mode, the detection solution will change with the different detection thresholds. If the threshold is too large, the velocity might also be reset during the swing phase, resulting in a reduced position update of each step after integration. Therefore, it is necessary to develop a method to produce adaptive threshold values for the zero velocity interval detection according to the motion modes. 

**Figure 5:**
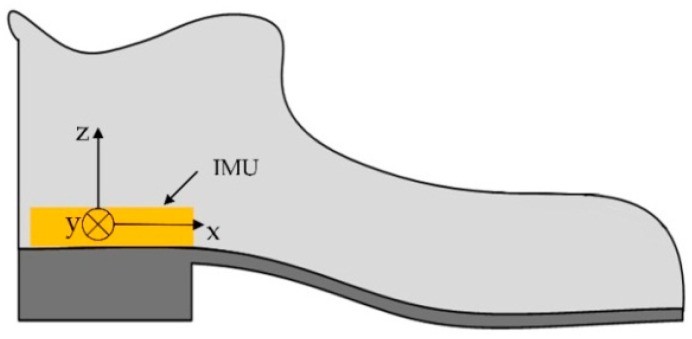
The foot-mounted inertial navigation system.

In inertial navigation systems, the IMU is installed in a shoe, as can be seen in Figure 5. Note that the *y*-axis is nearly perpendicular to the sagittal plane of the foot. Thus, when the user is walking or running, the dominant rotation axis is the *y*-axis. In our system, the *y*-axis gyroscope output has positive values when the foot is rotating clockwise. 

**Figure 6:**
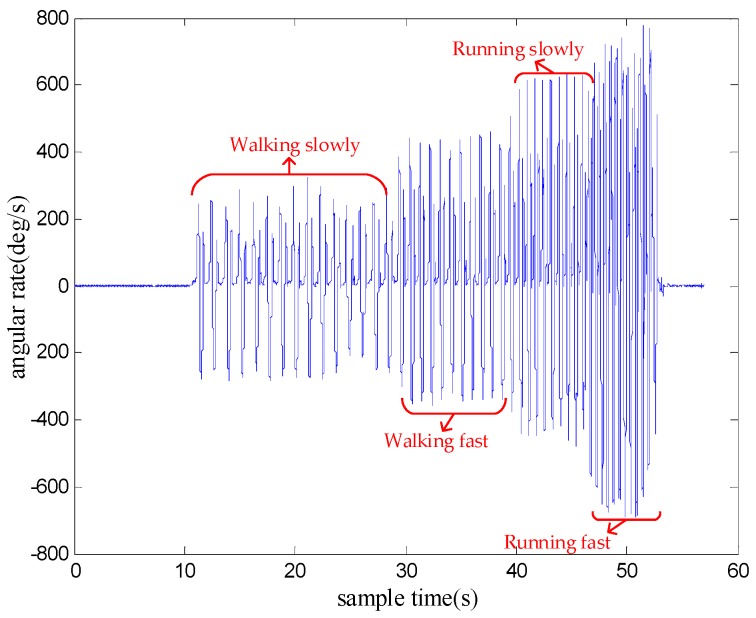
The *y*-axis gyroscope output of the four motion modes: walking slowly, walking fast, running slowly and running fast.

Figure 6 shows the *y*-axis gyroscope output of the four motion modes: waking slowly, walking fast, running slowly and running fast. It can be seen that the stronger and more intense the movement is, the larger the variation of angular rate magnitude. Therefore, it is possible to recognize and classify the motion modes by analyzing the magnitude of *y*-axis angular rate.

The performance of the ZUPT technique relies on the accuracy of zero velocity detection, which can further determine the final positioning errors. Hence, a feasible method to determine the adaptive thresholds is to analyze the positioning error corresponding to the given detection threshold. To this end, we asked the user to walk along the planned path with one motion mode at a constant speed and we collected the sensor data. We calculated the positioning errors under different thresholds and the threshold corresponding to the least positioning error was determined as the optimal detection threshold. Then, we changed the motion mode and repeated the experiment. 

**Figure 7:**
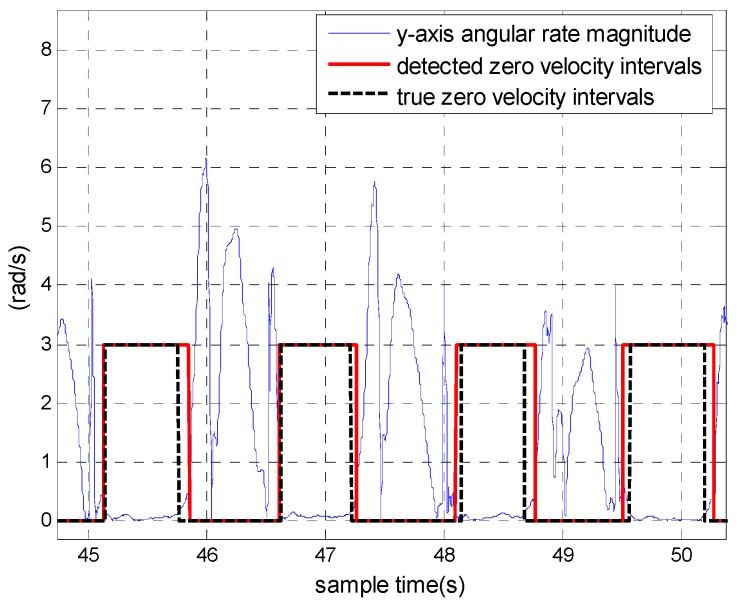
The zero velocity detection results when a pedestrian ascends stairs using the optimal detection threshold of level walking.

**Figure 8:**
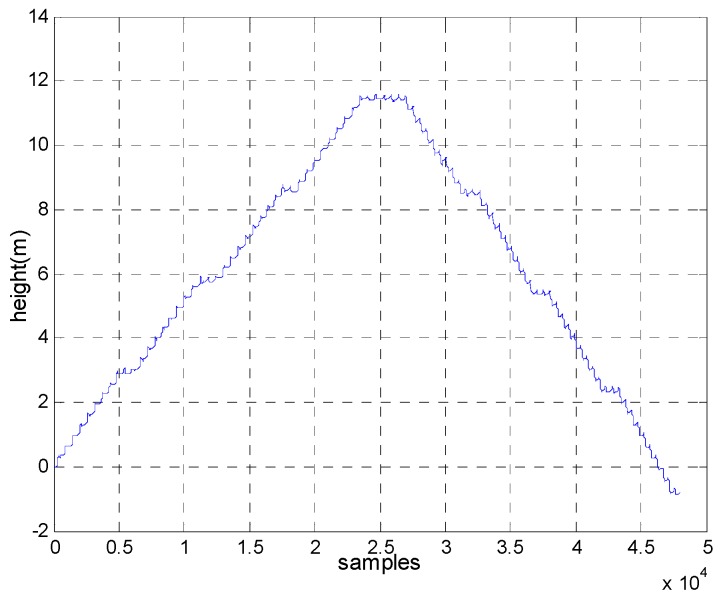
The height drift caused by incorrect zero interval detection.

Note that the pattern of the foot striking the ground when ascending stairs is different from that during level walking and the residual acceleration and angular rate during zero velocity intervals are much smaller in the former than the latter. If the optimal detection threshold of level walking was used to detect zero velocity when a pedestrian ascended the stairs, the accuracy of the detected zero velocity intervals would have been degraded, which could lead to height drift. This is demonstrated in Figure 7 and Figure 8. The pedestrian climbed from the first floor to the fourth floor and then returned to the starting point. Figure 7 shows the detected zero velocity intervals and Figure 8 depicts the height trajectories. The total height drift is about 1 m. Thus, it is necessary to distinguish ascending stairs and descending stairs from level walking.

**Figure 9:**
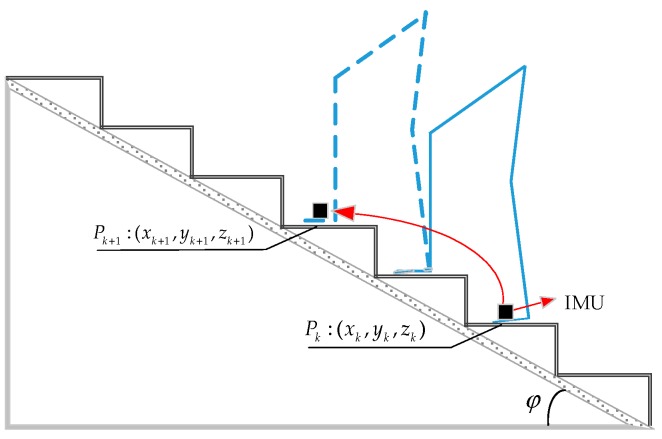
Sketch of ascending stairs.

Generally, the structure of stairs follows certain building codes and the inclination angle of stairs is typically between 20° and 50°, as shown in Figure 9. φ denotes the inclination angle of stairs. $P_{k}$ and $P_{k+1}$ represent the position of the kth and (k+1)th step, respectively. 

The inclination angle between $P_{k}$ and $P_{k+1}$ can be estimated by

(10) $$\eta_{k}=\arctan\left(\frac{\left|z_{k+1}-z_{k}\right|}{\sqrt{{\left(x_{k+1}-x_{k}\right)}^{2}+{\left(y_{k+1}-y_{k}\right)}^{2}}}\right)$$

$\eta_{k}$ is approximately zero when a person walks on flat ground and it is approximately the inclination angle φ when ascending stairs. The motion mode can be classified as climbing stairs if $\eta_{k}$ is greater than 25°and less than 50°. 

## 4. Results and Discussion

**Figure 10:**
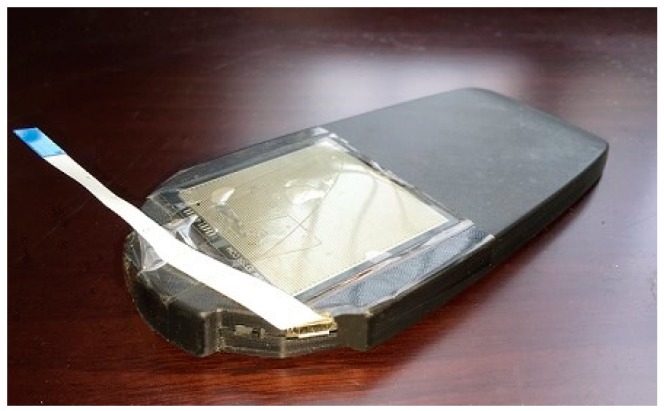
The insole-shaped MIMU module.

In this section, we discuss how the proposed method was verified using the experiment data acquired from a module developed by the researchers. This module was a multiple inertial measurement units (MIMU) platform with eight IMUs. The IMU that we employed was the MPU9250 (InvenSense Inc., San Jose, CA, USA), which included a tri-axis accelerometer and a tri-axis gyroscope. The pressure sensor we used was the FSR 406 (Interlink Electronics Inc., Camarillo, CA, USA). The output range of the pressure sensor was 0–3 V. The active area of FSR 406 was 38.1 × 38.1 mm and the nominal thickness was 0.46 mm. More details about the FSR 406 can be found in Reference [25]. The pressure sensor was attached to the upper surface of the positioning system, as shown in Figure 10. The sampling rate was configured at 400 Hz. The module was designed to fit in the insole-shaped shell, so it could be easily attached into shoes. 

### 4.1. Adaptive Threshold

To use the proposed detector effectively, there were several tuning parameters in Equation (9) that needed to be quantified. We calculated the test statistics using a data window size of N=7. According to the results of Reference [10], the noise standard deviations $\sigma_{a}$ and $\sigma_{\omega }$ were set to 0.01 m/s^2^ and 0.1 rad/s, respectively. $\sigma_{\rho }$ denotes the weight of information from the pressure sensor in the detector. If $\sigma_{\rho }$ is chosen to be too small, the detection accuracy of each zero velocity interval will be degraded. If the value of $\sigma_{\rho }$ is too large, the false-alarm probability will be increased. Hence, we set the noise standard deviation $\sigma_{\rho }$ to 0.001, which makes the information from the pressure sensor constitute about 25% of the total test statistics. 

This experiment was conducted by a pedestrian (here, we called him Person A), a 27-year-old male with a height of 1.73 m and a weight of 68 kg. The pedestrian walked along a square trajectory 6 m long for 10 loops under a certain motion mode. The pedestrian conducted six types of motion: walking slowly, walking normally, walking fast, running slowly, running normally and running fast. The average magnitude peaks of the *y*-axis gyroscope outputs were approximately 3.3, 5.1, 6.7, 8.6, 10.4 and 12.5 rad/s, respectively. 

**Figure 11:**
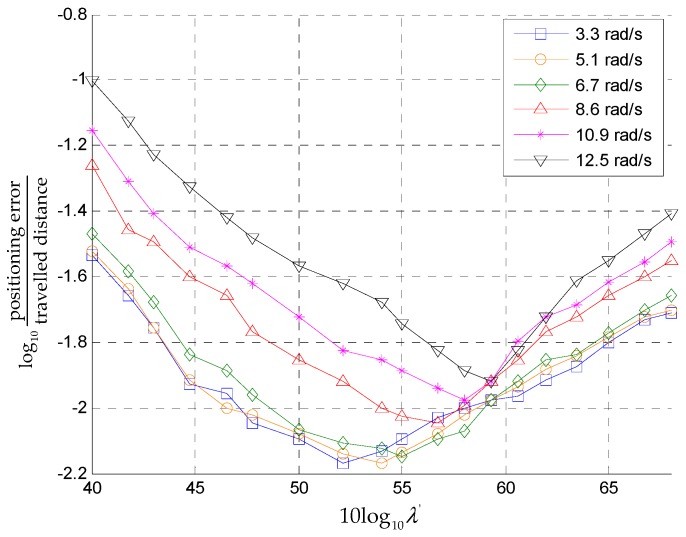
The positioning error as a function of the detection threshold.

For each motion mode, we calculated the positioning errors under different thresholds and the threshold corresponding to the least amount positioning error was chosen as the optimal threshold. Figure 11 shows the relationship between the positioning error and the detection threshold under six motion modes. 

We also conducted experiments on staircase movement with the aforementioned six types of motion and found the optimal threshold in the same way. Table 1 summarizes the optimal thresholds of different motion modes.

According to the results of Table 1, we determined the threshold functions based on second-order polynomial fitting as follows

(11) $$\begin{array}{l} f_{l}(\omega )=6232\omega^{2}-40063\omega \;\;+304998\\ f_{s}(\omega )=5582\omega^{2}-27456\omega \;\;+149359 \end{array}$$

where $f_{l}(\omega )$ represents the threshold function of level walking/running, $f_{s}(\omega )$ represents the threshold function of ascending/descending stairs and ω is magnitude peak of *y*-axis gyroscope output during a gait cycle.

### 4.2. Zero Velocity Interval Detection

**Figure 12:**
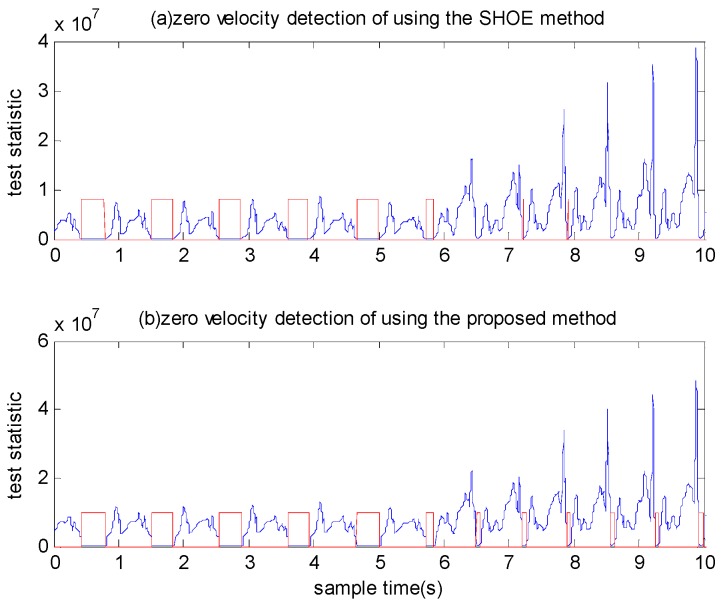
The zero velocity interval detection comparison between when a person is walking and running slowly.

**Figure 13:**
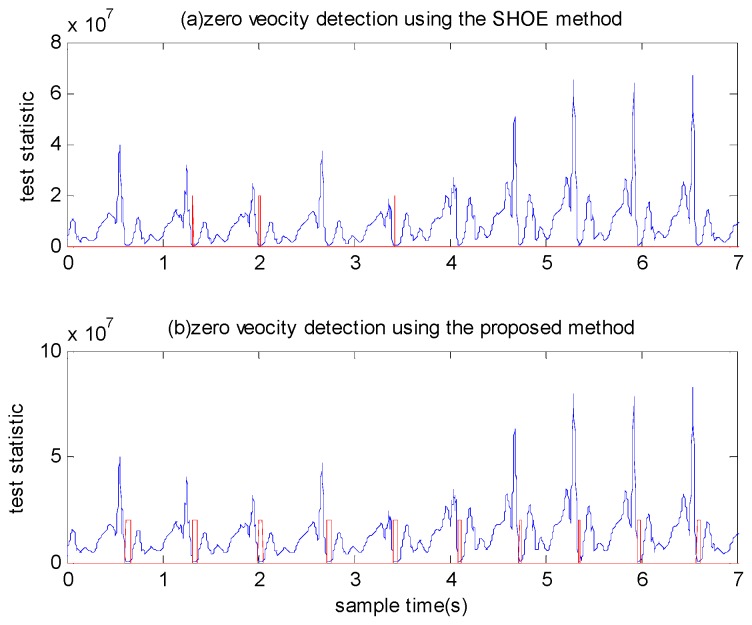
The zero velocity interval detection comparison between when a person is running slowly and running fast.

We calculated the test statistics using a window size of N=7. Three types of motion were conducted by Person A: walking normally, running slowly and running fast. To provide a comparison, we examined the performance of the SHOE detector using the same experimental data. The detected zero velocity intervals are shown in Figure 12 and Figure 13. The blue line denotes the test statistic and the red line denotes the detected zero velocity interval results. As illustrated in Figure 12, although the SHOE detector detected the zero velocity intervals correctly during walking, it did not perform well when the pedestrian ran slowly. In Figure 13, it is shown that the SHOE detector almost failed to detect the zero velocity intervals during fast running. In all three types of motion, the proposed method detected the zero velocity intervals correctly.

**Figure 14:**
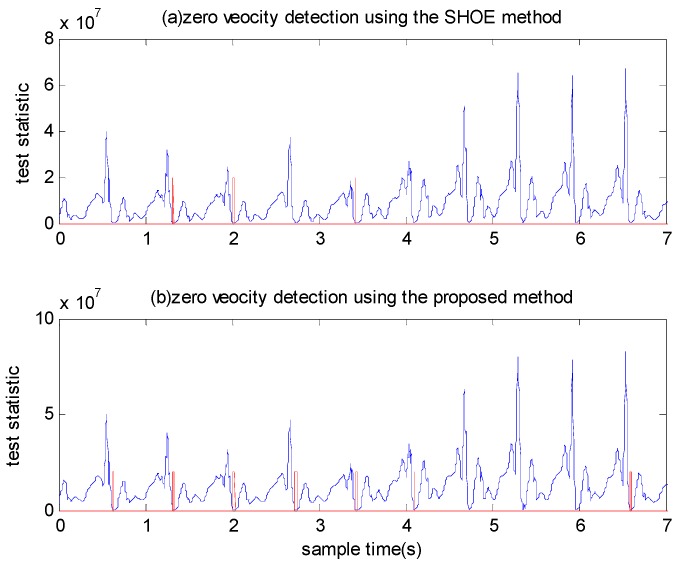
The zero velocity interval detection comparison using a fixed threshold for both detectors when a person is running slowly and running fast.

**Figure 15:**
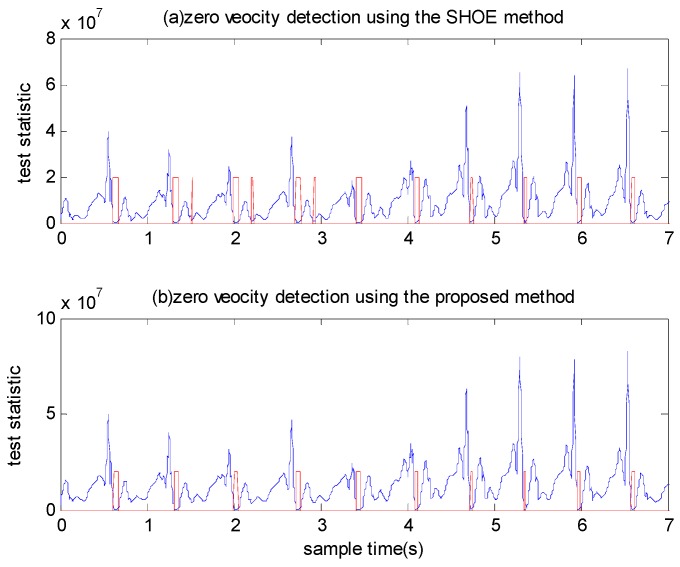
The zero velocity interval detection comparison using adaptive threshold for both detectors when a person is running slowly and running fast.

We also tested the performance of the SHOE detector and the proposed detector using a fixed threshold or adaptive threshold for both detectors. As shown in Figure 14, most zero velocity intervals cannot be detected by both detectors using a fixed threshold under running fast mode. In Figure 15, although the zero velocity intervals can be detected by the SHOE detector using adaptive threshold, it also makes some false alarm detection. The proposed detector performs better than the SHOE detector when using adaptive threshold.

### 4.3. Performance in Real Indoor Environments

**Figure 16:**
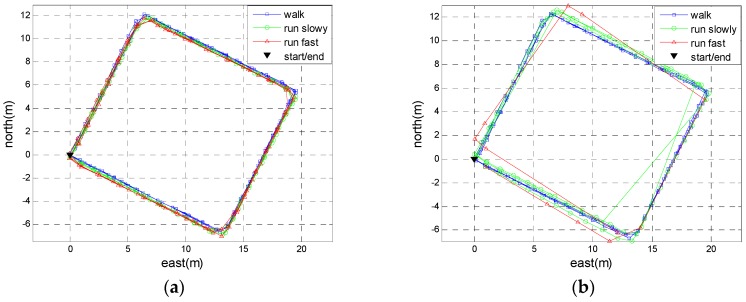
The step-wise trajectories of Person A. (**a**) The trajectories using the proposed method. (**b**) The trajectories using the SHOE method.

**Figure 17:**
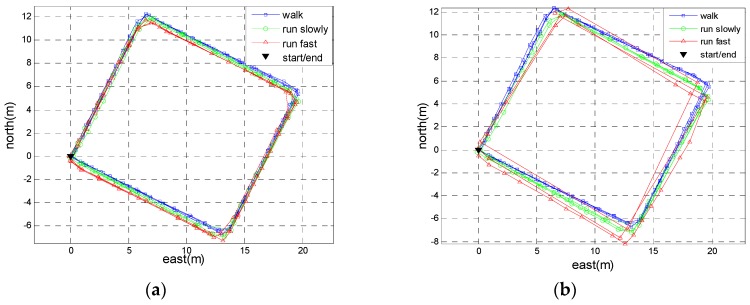
The step-wise trajectories of Person B. (**a**) The trajectories using the proposed method. (**b**) The trajectories using the SHOE method.

Two experiments were conducted in real environments to verify the performance of the proposed method. In the first experiment, Person A walked along the side-lines of half a basketball court (15 m long and 14 m wide), then ran slowly and ran fast along the same path. Each motion mode repeated for three loops and the total travelled distance was approximately 522 m. In order to evaluate the generality of the adaptive threshold determined by Person A, another person (here, we called him Person B)—a 29-year-old male with a height of 1.79 m and a weight of 75 kg—repeated this experiment using the same positioning system. We calculated the trajectories using the proposed method and the SHOE method. Figure 16 shows the trajectories of Person A and Figure 17 shows the trajectories of Person B. Table 2 summarizes the final positioning errors along the four trajectories. From Table 2, it can be observed that the performance of the proposed method is better than the SHOE method in running mode. Moreover, the performance of the proposed method differs between Person A and Person B. The adaptive threshold determined by Person A would not be optimal for other persons because everyone’s motion characteristic is unique. Even so, the positioning accuracy of the proposed method for Person B outperforms the SHOE method.

**Figure 18:**
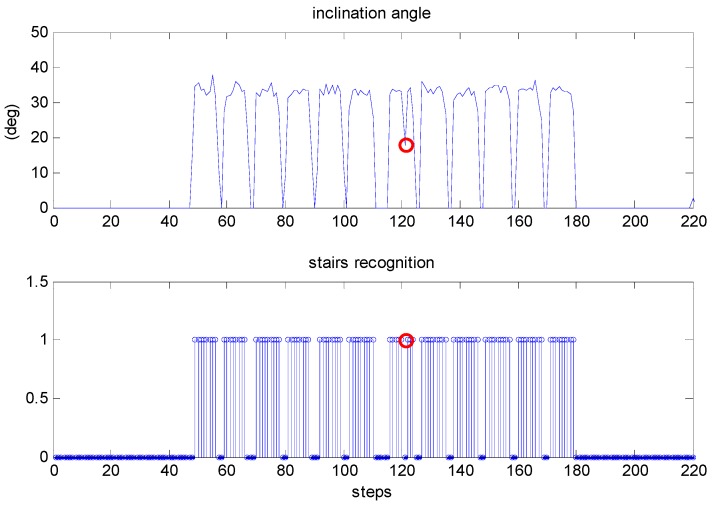
The inclination angle of the trajectory and the result of stairs recognition.

We also conducted a test along another trajectory that included staircase movement to evaluate the performance of the proposed method in stairs recognition. The pedestrian walked out of a room and climbed from the first floor to the seventh floor, then returned to the start point along the same route. The travelled distance and the height of the stairs were approximately 273 and 18 m, respectively. Figure 18 shows the inclination angle of the trajectory and the result of the stairs recognition. Only one step of staircase movement was not detected. 

**Figure 19:**
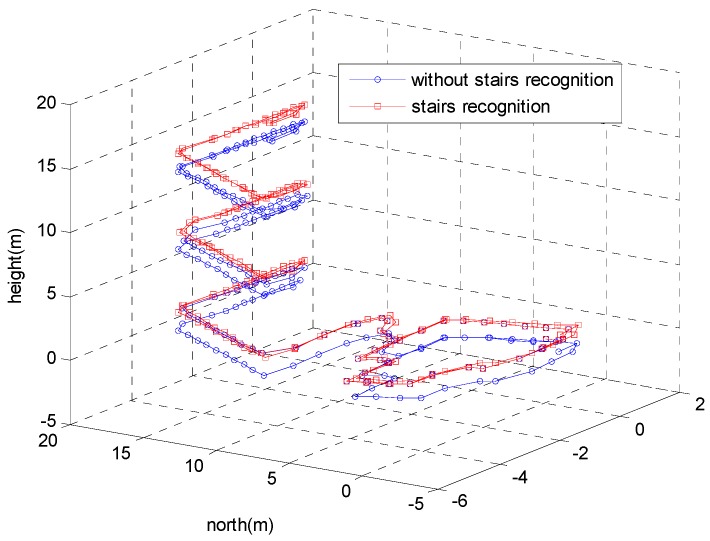
The 3D trajectories of the proposed method with stairs recognition versus without stairs recognition.

**Figure 20:**
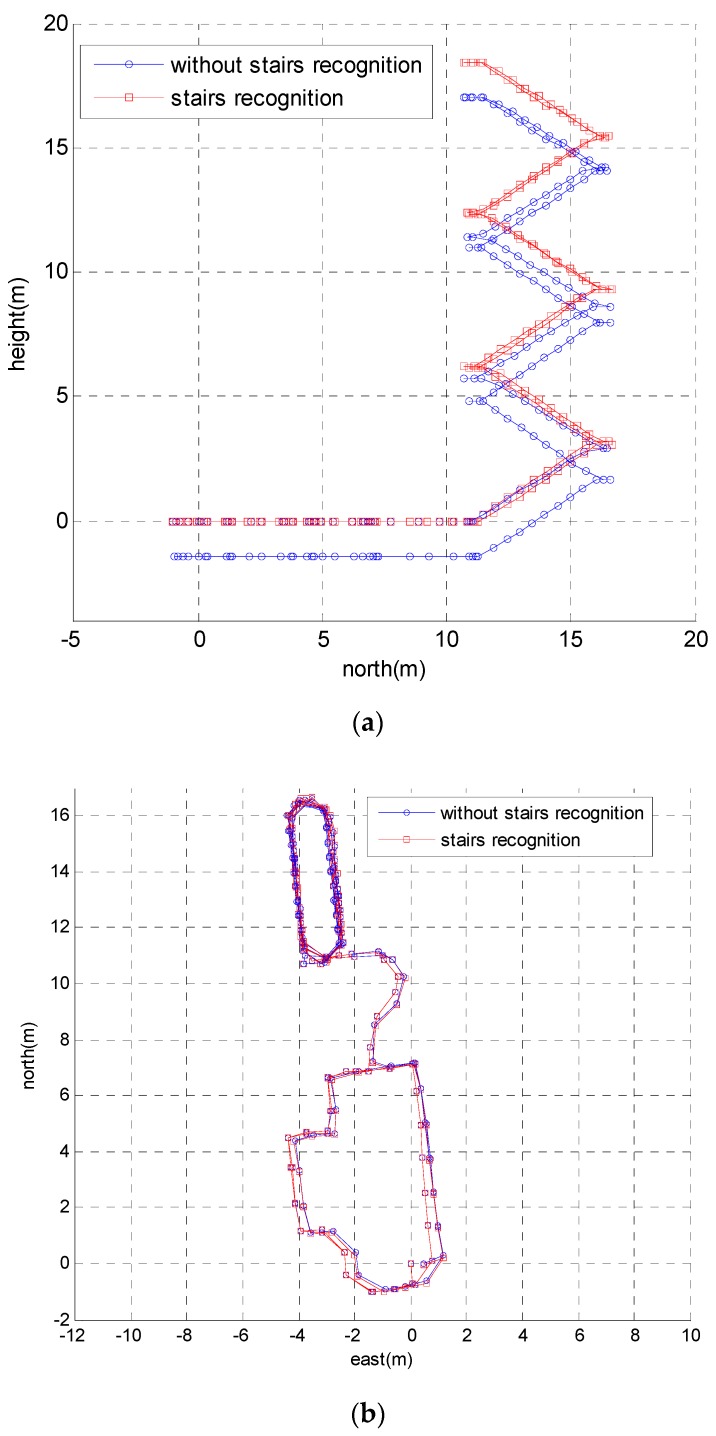
The 2D trajectories of the proposed method with stairs recognition versus without stairs recognition. (**a**) The calculated trajectories in the *y*–*z* plane. (**b**) The calculated trajectories in *x*–*y* plane.

Figure 19 depicts the 3D trajectories of the proposed method with stairs recognition versus without stairs recognition. The height error of the proposed method without stairs recognition was approximately 1.5 m. The height error was reduced to 0.1 m by adopting stairs recognition. Figure 20 shows the 2D trajectories of the test. As shown in Figure 20b, the trajectories in the *x*–*y* plane of the two methods were almost the same, which indicates that stairs recognition only affected the height of the trajectory. The trajectories in the *x*–*y* plane were not exactly equal because the ZUPT algorithm cannot estimate heading errors caused by gyroscope biases, which are a primary error source for ZUPT-aided pedestrian navigation system (PNS).

## 5. Conclusions 

This paper presents an adaptive zero velocity detection method for the ZUPT-aided PNS. The information extracted from the accelerometer, gyroscope and pressure sensor were fused to construct a zero velocity detector. The difference of the test statistics of the detector between walking and running were reduced during zero velocity intervals. To improve the accuracy of each zero velocity interval, the magnitude peak of the *y*-axis gyroscope was used to classify the motion mode and determine the adaptive threshold for the detector. In addition, a stairs recognition method was developed to distinguish staircase movement and eliminate the height drift. The experiment results show that the proposed method can detect zero velocity correctly under various motion modes and perform well in real indoor scenarios. 

## Figures and Tables

**Table 1:** The optimal thresholds of different motion modes.

**Level Walking/Running**	**3.3 (rad/s)**	**5.1 (rad/s)**	**6.7 (rad/s)**	**8.6 (rad/s)**	**10.9 (rad/s)**	**12.5 (rad/s)**
optimal threshold	$2.45\times 10^{5}$	$2.62\times 10^{5}$	$3.13\times 10^{5}$	$4.24\times 10^{5}$	$5.76\times 10^{5}$	$7.82\times 10^{5}$
**Stairs Ascending/Descending**	**3.5 (rad/s)**	**5.4 (rad/s)**	**7.1 (rad/s)**	**8.7 (rad/s)**	**9.8 (rad/s)**	**11.6 (rad/s)**
optimal threshold	$1.27\times 10^{5}$	$1.56\times 10^{5}$	$2.30\times 10^{5}$	$3.25\times 10^{5}$	$4.31\times 10^{5}$	$5.63\times 10^{5}$

**Table 2:** The final positioning errors along the four trajectories.

		Positioning Error/Travelled Distance (%)			
		Walking	Running Slowly	Running Fast	Total
Person A	Proposed method	0.21	0.25	0.28	0.25
	SHOE	0.27	0.82	1.87	0.98
Person B	Proposed method	0.26	0.39	0.57	0.41
	SHOE	0.25	0.73	1.83	0.94
